# Cerebro-Spinal Meningitis

**Published:** 1877-03

**Authors:** J. G. Westmoreland

**Affiliations:** Prof. of Materia Medica and Therapeutics in Atlanta Medical College, Atlanta, Ga.


### CEREBRO-SPINAL MENINGITIS.

By J. G. WESTMORELAND, M.D.,
Prof. of Materia Medica and Therapeutics in Atlanta Medical College, Atlanta, Ga.

The generally fatal termination of violent cases of the disease whose name heads this article, necessarily awakens professional and public interest on the subject. Humanity calls for unprejudiced, thorough investigation, on the part of medical men, of the true pathology, in order to the institution of successful treatment. Opposite theories have been promulgated, and upon them, of course, opposite plans of treatment are adopted. One, at least, of these must be incorrect, and must lead to failure of success, if not to positive injury.

The medical profession is unequally divided on this subject; the larger portion favoring the asthenic or adynamic pathological theory, which recognises only depressed vital functions, caused by the direct poisonous impression of some unknown agent upon the nervous centres, without the existence, necessarily, of any local physical lesion. They cite, in proof of this, instances of sudden alarming prostration in an hour after the attack, and before sufficient time has been had for the establishment of true inflammatory action. The postmortem evidences of inflammation are considered referable to incidental lesions of the meninges that occur during the progress of, and resulting from the poisoned condition of the neurine or brain substance which they envelop.

The treatment pursued by this class of pathologists must necessarily consist of means calculated to excite temporarily, and invigorate permanently, the great central organs of vital force. Hence, reliance is had by them, in cardiac, brain and spinal stimulants and tonics, quinine being considered an essential permanent invigorator.

On this, as well as some other pathological questions, we have for many years been alligned with a minority of the profession. We are unfortunate in not being able to reconcile the symptoms, result of treatment and postmortem appearances, with the adynamic theory of cerebro-spinal meningitis. It is particularly a source of regret, since a decided preponderance of distinguished medical authorities favors this view of the subject.

While the initiatory symptoms, as well as those often found in avanced stages of the disease, show great reduction of vital power, there is nothing in them inconsistent with the effect that may be expected during any stage of inflammation in a vital organ, or in a part contiguous to it. Particularly is this true of the brain and other nervous centres. The function may be disturbed, partially or perfectly, by a poison affecting the organic tissue. The same may be the effect of a redundancy or deficiency of blood in the organ; the latter leading to inactivity from a want of the accustomed physiological excitant, the former from unusual mechanical pressure.

The first stage of inflammation is characterized by determination of blood to, or congestion in, a part which immediately succeeds the irritation set up, or the general circulatory derangement upon which congestion depends.

Now, with these facts before us, it is not at all unreasonable to expect depression of the vital powers, in degree proportionate to the superabundant amount of blood in the meninges, mechanically pressing unduly the organ within. In this way symptoms of great nervous prostration may be the first evidence of disease exhibited in the beginning of a highly phlogistic or inflammatory condition. This state of depression, from which, when the cause is excessive and unremoved, the patient sometimes fails to react, is the main cause of opposition to the inflamation theory, and to the antiphlogistic plan of treatment. Practitioners who have nearly lost sight of depletion, even in a bounding state of circulation, look with horror at the abstraction of blood from a semi-conscious subject with cool skin and feeble circulation.

It is generally admitted that autopsies made of this disease reveal evidences of engorgement, fully established inflammation or the results of this process, according to the state of progress at the time of death. Statistics not only show this, but when made up properly, fairly and honestly, go very far toward settling pathological and thereapeutical questions on this and other medical subjects.

In 1873 the author of this article had the honor of reading before the Medical Association of Georgia, a paper entitled "Cerebro-Spinal Meningitis—its pathology and treatment"—in which will be found reports of the disease in this city the previous winter, coming under the notice of two physicians only. (Transactions of 1873, page 132). In order to more decisive results the statistics should be extended, including the experience of a larger number of practitioners. To this end we propose, at some future time, to compile, from such sources as we may be able to obtain them, statistics of the disease, showing the comparative mortality under the quinine supporting treatment and the antiphlogistic plan. Since the five cases were reported to the Medical Association, we have had charge of three others. All of these were treated by prompt and decided depletion, opium, counter-irritation and large doses of calomel. Two recoveries and one death occurred; and fairness compels us to say that the failure of success followed no less vigorous antiphlogistic treatment than that had in the cases of recovery.

This article was commenced with the view of having the table of statistics to accompany it, but so much difficulty was met with in getting up correct reports from physicians, that it is thought proper to defer the publication for the present. A table made up on the basis of "antiphlogistic" and "supporting" treatment was found to be erroneous, on account of the different interpretation of these terms by those from whom reports were obtained. One physician considers opium, calomel, quinine and counter-irritants, antiphlogistic means, while another calls them supporting agents. For example, a report of ten cases was furnished us by a physician of the city, in which the treatment, as I learned, consisted chiefly of quinine, leeches and blisters. This he denominated antiphlogistic treatment, and placed the nine deaths and one recovery under this head. General and local blood-letting, opium, large doses of mercury and active cathartics, we think are more appropriate antiphlogistic means for the treatment of acute inflammation of parts connected with vital organs, which it is important to arrest before fatal effects are produced. Such we consider the disease in question, and hence the importance of prompt and decided treatment.

